# The Knowledge-Sharing Implications of Social Responsibility of Firms: The Importance of Ethical Climate

**DOI:** 10.3390/bs13070608

**Published:** 2023-07-21

**Authors:** Yunsook Hong, Byung-Jik Kim, Min-Jik Kim

**Affiliations:** 1 College of Business, University of Ulsan, Ulsan 44610, Republic of Korea; yhong@ulsan.ac.kr; 2 School of Industrial Management, Korea University of Technology and Education, Cheonan 31253, Republic of Korea

**Keywords:** corporate social responsibility, ethical climate, perceived organizational support, organizational commitment, knowledge-sharing behavior

## Abstract

Using a variety of theoretical foundations, this paper examines the association between corporate social responsibility (CSR) and employees’ knowledge-sharing behavior, investigating the sequential mediation role of perceived organizational support and organizational commitment as well as the moderating role of ethical climate. Hypotheses were formulated to address both the sequential mediation effect of perceived organizational support and organizational commitment on the link between CSR and knowledge-sharing and the moderation effect of ethical climate on the relationship between CSR and perceived organizational support. In order to mitigate the problems of potential common method bias, data were gathered from a sample of 204 South Korean employees at three distinct time points. The results of the study offer compelling evidence that CSR activities exert a positive influence on employees’ knowledge-sharing behavior, an effect that is sequentially mediated by both perceived organizational support and organizational commitment. Furthermore, the research uncovers the positive moderating role played by the ethical climate in the relationship between CSR and perceived organizational support. By integrating multiple theoretical frameworks, this study not only advances the extant literature but also offers invaluable insights for organizations striving to bolster knowledge-sharing through the strategic implementation of CSR initiatives.

## 1. Introduction

In recent times, corporate ethics has emerged as a crucial aspect for businesses, spurring extensive exploration by both academics and practitioners alike in the realm of corporate social responsibility (CSR) [1,2,3]. CSR encompasses a diverse array of interpretations and can be generally defined as the aggregation of strategies and practices implemented by organizations to augment their economic, social, and environmental performance while concurrently addressing the multifaceted needs of stakeholders, such as employees, consumers, local communities, governments, and the environment [4,5]. Although numerous investigations have been conducted to analyze the impact of CSR on organizational performance, the outcomes have remained ambiguous [6,7,8,9]. Certain studies propose that CSR initiatives function as a strategic “investment,” offering organizations a competitive edge [10,11,12]. Conversely, some scholars have argued that allocating resources toward social responsibilities may be tantamount to a “cost” that can impede operational effectiveness [13,14]. In an attempt to harmonize these divergent viewpoints, researchers have examined various underlying mechanisms and situational factors that moderate the relationship between CSR and organizational outcomes [4,6,9,15,16,17,18]. Despite numerous inquiries into the effect of CSR on organizational outcomes, various gaps persist within the current literature [6,7,8].

First, although earlier research has delved into the ramifications of CSR on organizational members, the majority of these studies have primarily focused on individuals’ perceptions and attitudes. These encompass various aspects, such as identification with their organization, organizational commitment, job satisfaction, organizational trust, work meaningfulness, intrinsic motivation, and employee engagement [19,20,21,22,23,24,25,26]. Conversely, there has been a comparatively limited focus on employee behaviors [6,7,8,27]. Although perceptions and attitudes represent essential outcomes at the individual level within an organization, they are likely to ultimately manifest themselves in behaviors [6,7,27]. Consequently, we postulate that employee behavior exhibits a stronger association with various organizational outcomes in comparison to perceptions and attitudes, rendering it imperative to investigate the impact of CSR on member behaviors.

Second, previous research has insufficiently investigated the impact of CSR on employee knowledge-related behaviors [6,7,27]. While research has established the positive influence of CSR on various organizational outcomes, the impact of CSR on employees’ knowledge-sharing behavior remains underexplored. Knowledge-sharing is a critical determinant of innovation and organizational performance [27,28]. Owing to the considerable impact that knowledge possesses in generating value-added products and services, which subsequently exerts a substantial effect on a firm’s competitive advantage [29,30], exploring how CSR influences employees’ knowledge-related behaviors is essential [31,32]. In light of the significance of knowledge-sharing behavior, it is essential to investigate CSR’s influence on knowledge-sharing behavior.

Third, relevant to the second research gap in existing CSR literature, there has been an insufficient examination of the mediators and moderators that impact the links between CSR and knowledge-related behaviors [6,7,27]. As recognizing the mediators and moderators can provide a comprehensive understanding of this connection [6,7], it is crucial to explore the mediating and moderating variables in the CSR knowledge-related behaviors link.

To address these research gaps, this study investigates the underlying procedures (mediators) and contextual variables (moderators) present in the link between CSR and knowledge-related behaviors. Among various knowledge-related behaviors, we focus on knowledge-sharing behavior, which is defined as the extent to which an employee disseminates knowledge, encompassing the act of exchanging information, skills, expertise, and knowledge among individuals or groups within an organization [33]. The profound significance of knowledge-sharing in the contemporary business landscape cannot be understated. This important practice, as elucidated by Wang and Noe [33], encompasses the dissemination of information, skills, expertise, and knowledge among employees or groups within an organization. When successfully implemented, knowledge-sharing cultivates an environment conducive to continuous learning, innovation, and growth, consequently empowering organizations to capitalize on their intellectual assets and achieve a sustainable competitive advantage [30].

This paper proposes that CSR can increase the quality of employee knowledge-sharing behavior through the sequential mediating effect of perceived organizational support (POS) and organizational commitment (OC); in other words, CSR would sequentially increase the degree of POS and OC, which ultimately enhances the extent of knowledge-sharing behavior. Additionally, an ethical climate would function as a positive moderator in the link between CSR and POS.

Our purpose is to find the mechanisms through which CSR affects employees’ knowledge-sharing behavior. The contributions of this study are as follows. First, by exploring the influence of CSR on knowledge-sharing behavior, it expands the understanding of the factors that encourage employees to engage in knowledge-sharing activities within organizations. Second, by examining the mediating roles of POS and organizational commitment, this study offers an elaborate view of the underlying mechanisms through which CSR influences employee knowledge-sharing behavior, enriching the theoretical underpinnings of the CSR literature. Third, by investigating the moderating effect of ethical climate, the study highlights the significance of fostering an ethical work environment to enhance the impact of CSR on employee perceptions and behavior. Lastly, the rigorous methodology employed in this study, involving a longitudinal survey design with a diverse sample of Korean panelists, lends credibility to its findings and their implications for both research and practice. By shedding light on the critical mechanisms linking CSR initiatives with employee knowledge-sharing behavior, this research offers valuable insights for organizations aiming to foster a collaborative and innovative work environment through socially responsible practices.

By investigating these relationships in a South Korean context and using a longitudinal research design with three time points, this study contributes to the generalizability of the findings and offers implications for both researchers and practitioners in the field of CSR, organizational behavior, and human resource management.

## 2. Theory and Hypotheses

### 2.1. CSR and Knowledge-Sharing Behavior

To the best of our knowledge, although few empirical studies to date have examined the influence of CSR on knowledge-sharing behavior [6,7,8], the relationship can be explained through several theoretical foundations, including social exchange perspective, social identity perspective, organizational citizenship behavior perspective, and social cognitive perspective. These theories provide a comprehensive understanding of the relationship between an organization’s CSR initiatives and its employees’ willingness to engage in knowledge-sharing behavior.

First, the social exchange theory posits that, when one party provides help or advantages to another, the recipient is inclined to reciprocate similarly [34,35,36]. Employees are among an organization’s primary stakeholders and are both directly and indirectly affected by its CSR practices [6,7,27]. Therefore, they are likely to feel an obligation toward their company, ultimately expressing their gratitude through positive actions, which can include exhibiting knowledge-sharing behavior [6,7,30]. An organization’s CSR initiatives also encourage employees to share the knowledge they have acquired in their profession with fellow employees with a harmless and supportive purpose.

Second, the social identity theory, proposed by Tajfel and Turner [37], suggests that individual members tend to categorize themselves into groups based on shared attributes, deriving self-esteem from their group membership. CSR initiatives play a pivotal role in fostering a positive organizational identity by demonstrating the organization’s commitment to addressing social, environmental, and ethical issues. Employees who perceive their organization as actively engaged in CSR endeavors are inclined to have heightened pride in their affiliation with their company; this pride, in turn, cultivates a sense of trust and collaboration among employees, thereby promoting knowledge-sharing behavior.

Third, organizational citizenship behavior theory, expounded by Organ [38], delineates voluntary actions undertaken by employees that surpass their formal job requirements, contributing to the organization’s overall functioning and success. CSR initiatives can act as a catalyst, inspiring employees to engage in organizational citizenship behaviors, such as knowledge-sharing, by fostering a sense of shared values, trust, and commitment to the organization. Employees, perceiving their organization as dedicated to CSR, may exhibit a proclivity to reciprocate by engaging in knowledge-sharing behavior, driven by a sense of responsibility to contribute to their organization’s success.

Fourth, social cognitive theory, introduced by Bandura [39], asserts that human behavior is shaped by the reciprocal interaction of personal factors, environmental factors, and behaviors. CSR initiatives can influence employees’ knowledge-sharing behavior by providing a supportive organizational context and promoting a culture of collaboration and learning. Employees, perceiving their organization as actively engaged in CSR initiatives, may develop positive beliefs and expectations regarding the organization’s values and objectives, subsequently encouraging knowledge-sharing behavior.

In summary, the impact of CSR on employee knowledge-sharing behavior can be illuminated through the lenses of various theories. Employees who perceive their organization as actively engaged in CSR initiatives are more likely to participate in knowledge-sharing behavior, as they take pride in their association with the company, feel inspired to contribute to the organization’s success, and find themselves supported by a collaborative and learning-oriented organizational culture. Among the various theoretical perspectives that exist, this paper mainly focuses on the social identity perspective and social exchange perspective.

**Hypothesis 1.**  *CSR activities increase knowledge-sharing behavior by members*.

### 2.2. CSR and POS

We propose that CSR would increase the extent of employees’ POS [6,7]. POS constitutes a critical aspect of the employee–organization relationship. Fundamentally, POS refers to the extent to which employees discern that their organization not only appreciates their contributions but also exhibits genuine concern for their welfare [40]. In a broader sense, POS gauges an employee’s perception of the organization’s commitment to their success, satisfaction, and the attainment of their goals [40,41]. Indeed, the multifaceted nature of POS renders it a vital factor in fostering a supportive work environment. High levels of POS have been linked to a plethora of positive outcomes. Among these outcomes, one can enumerate enhanced job satisfaction, bolstered organizational commitment, and diminished turnover rates [42,43,44].

CSR activities are likely to convey an organization’s efforts to enhance the well-being of not only external stakeholders, such as clients, suppliers, and the environment, but also internal stakeholders, which include employees. By recognizing and addressing the interests of a diverse array of stakeholders, organizations cultivate a sense of trust and support among employees, who perceive that their organization values their welfare and appreciates their contributions.

Specifically, the influence of CSR on employees’ POS can be explained through the social identity theory. The social identity theory posits that individuals derive a sense of self and belonging from their membership in social groups [37]. Organizations engaging in CSR activities can shape employees’ social identity by enhancing their perceptions of the organization’s values and image. CSR initiatives contribute to employees’ positive perceptions of organizational support by fostering their sense of belonging to a socially responsible organization. As employees identify with their organization’s values and image, they perceive higher levels of organizational support, believing that their organization is committed to their well-being and long-term success.

**Hypothesis 2.**  *CSR activities may increase employee POS*.

### 2.3. POS and Organizational Commitment

The influence of employees’ POS on their commitment to their organizations can be explained through a theoretical foundation, such as the social exchange theory.

The social exchange theory, put forth by Blau [45], asserts that interpersonal relationships are governed by the reciprocity principle, which contends that individuals partake in exchanges to procure benefits from their counterparts. When employees receive support, resources, and acknowledgment from their organization, they are more predisposed to exhibit heightened commitment as a form of reciprocation [34,35,36]. Employees are likely to form a general perception of the extent to which their organization values their contributions and cares about their well-being. High levels of POS contribute to employees’ sense of obligation and desire to repay their organization in the form of exhibiting positive attitudes or perceptions such as organizational commitment. Moreover, POS fosters a sense of belonging and attachment to the organization, leading to increased affective commitment, which is one of the three dimensions of organizational commitment identified by Meyer and Allen [46].

**Hypothesis 3.**  *Increased POS may increase employees’ organizational commitment*.

### 2.4. Organizational Commitment and Knowledge-Sharing Behavior

This paper suggests that employees’ organizational commitment will enhance the extent of their knowledge-sharing behavior. The influence of organizational commitment on knowledge-sharing behavior can be bolstered through social exchange theory, which offers valuable insights into how an employee’s dedication to their organization influences their willingness to participate in knowledge-sharing behavior.

As mentioned, social exchange theory emphasizes the principle of reciprocity in social interactions, asserting that individuals engage in exchanges to reap benefits from others. When applied to organizational commitment, this theory suggests that employees exhibiting strong loyalty and attachment to their organization are more inclined to share knowledge. This propensity stems from their perception of their organization as a source of support, recognition, and growth opportunities, which in turn generates a sense of obligation to reciprocate by sharing knowledge [34,35,36]. Ultimately, this reciprocity bolsters the organization’s overall performance and competitive edge.

In addition, delving into the concept and its sub-dimensions of organizational commitment, Meyer and Allen [46] delineate three distinct dimensions of organizational commitment: affective, continuance, and normative. Affective commitment pertains to an employee’s emotional connection to the organization, while continuance commitment encompasses the employee’s perceived costs associated with leaving the organization. Conversely, normative commitment entails an employee’s obligation to remain with the organization. Employees demonstrating high levels of affective and normative commitment are more prone to engage in knowledge-sharing behavior, as they more closely align with the organization’s objectives and sense a responsibility to contribute to its accomplishments. Based on the above arguments, we propose the following hypothesis.

**Hypothesis 4.**  *Increased organizational commitment may increase knowledge-sharing behavior*.

### 2.5. Sequential Mediation of POS and Organizational Commitment in the Link between CSR and Knowledge-Sharing Behavior

The current study proposes that the connection between CSR and knowledge-sharing may be sequentially mediated by POS and organizational commitment. This mediatory structure is based on the context–attitude–behavior framework, as demonstrated in prior research [47,48]. This framework posits that an organization’s contextual and environmental components, including its systems, practices, regulations, and climates, significantly influence the attitudes and behaviors of its members. As a crucial contextual element, CSR affects employees’ attitudes, specifically concerning POS and organizational commitment, which in turn impacts their behaviors, such as knowledge-sharing behavior. Therefore, this study argues that the sequential mediating roles of POS and organizational commitment enable the effect of CSR on employee knowledge-sharing behavior.

**Hypothesis 5.**  *The relationship between CSR and knowledge-sharing behavior is sequentially mediated by POS and organizational commitment*.

### 2.6. Moderation of Ethical Climate in the Link between CSR and POS

As mentioned above, the argument that CSR activities would lower employees’ POS may be reasonable. However, it might be overly reductive to consider this connection as universally applicable [6,7]. The current investigation posits that the link between CSR and POS may fluctuate based on employee attributes and organizational contexts. Using social identity and social exchange theories, we attempted to propose that an ethical climate could serve as an amplifier that positively moderates the link between CSR and POS. An organization’s ethical climate influences employees’ ethical behavior, decision-making, and attitudes toward their organization. In particular, an ethical climate that emphasizes CSR and value-based decision-making can strengthen the connection between CSR and POS. When employees perceive their organization as having a strong ethical climate, they are more likely to trust the motives behind CSR initiatives and feel that their organization genuinely cares about the well-being of various stakeholders, including them. These trust and belief in the organization’s motives enhance the link between CSR and POS.

More specifically, the moderation effect of ethical climate on the relationship between CSR and employee POS can be comprehensively explained by drawing upon various theoretical foundations, including the social identity theory and social exchange theory.

First, the social identity theory, posited by Tajfel and Turner [37], contends that individuals classify themselves into groups based on shared attributes and derive self-esteem from their membership within these groups. CSR initiatives contribute to the development of a positive organizational identity by demonstrating the organization’s commitment to addressing social, environmental, and ethical concerns. An ethical climate, characterized by a strong emphasis on ethical values and decision-making, reinforces the organization’s positive identity. In the presence of a robust ethical climate, employees are more inclined to perceive CSR endeavors as authentic efforts rather than mere public relations tactics. This perception augments their sense of organizational support, thereby elevating their POS.

Second, social exchange theory again postulates that social interactions are underpinned by the principle of reciprocity, wherein individuals engage in exchanges to procure benefits from others. An ethical climate establishes an atmosphere of trust and equity within an organization, which encourages employees to reciprocate through heightened commitment and support [34,35,36]. As organizations demonstrate CSR, employees perceive the company as being concerned for the welfare of a diverse range of stakeholders, including employees themselves. The existence of an ethical climate within the organization further bolsters this perception, as employees believe that their organization’s actions are guided by moral principles. This perceived support thus intensifies the relationship between CSR and POS.

**Hypothesis 6.**  *An ethical climate may positively moderate the link between corporate social responsibility and POS*.

**Figure 1:**
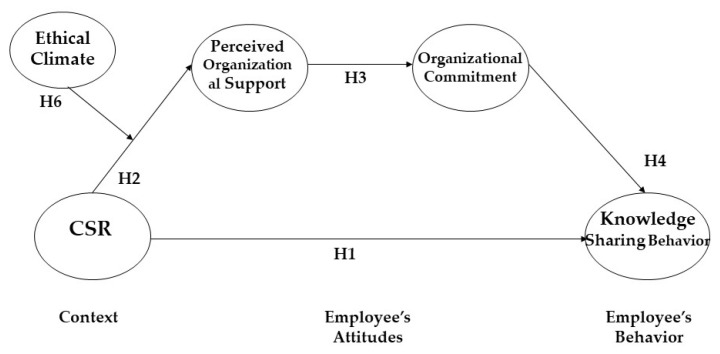
Theoretical model of this study.

Figure 1 illustrates our theoretical model and all six hypotheses formulated in this section.

## 3. Research Methodology

### 3.1. Participants and Procedure

In order to empirically examine the proposed theoretical framework, survey information was collected from South Korean employees aged ≥ 20 years through an online questionnaire platform. The study collaborated with a South Korean research institution that boasts the most comprehensive pool of Korean panelists. In order to reduce sampling bias, the research company chose participants randomly. To avoid same-source bias, data collection from respondents occurred during three separate time intervals, each spaced 4–6 weeks apart. The online questionnaire system enabled the tracking of respondents and the maintenance of their participation consistency from the first to the third stages. The time interval between the first and second surveys was 4–5 weeks, whereas the gap between the second and final surveys was extended to 5–6 weeks.

The research firm applied stratified random sampling to select participants, aiming to minimize potential sampling bias. This method involved choosing a random sample from each stratum, lessening the risks of biases related to various employee characteristics (e.g., gender, age, position, education, and industry type) that could potentially influence the results. Specialists from the research company contacted potential respondents, requesting their voluntary participation in the survey and assuring the confidentiality of their responses, which would be used exclusively for research objectives. The firm also ensured that informed consent and ethical compliance were obtained from those who agreed to take part and share their opinions. Respondents were offered a financial incentive of USD 7 from the research company.

During Time Point 1, 398 employees finished the survey, while 273 workers completed the survey at Time Point 2, and 207 employees completed the survey at Time Point 3. After accounting for missing data, the final analysis used data from 204 participants who completed all three surveys (response rate, 51.26%). The sample size determination was guided by prior research. To confirm the adequacy of the sample size, the minimum sample size was computed using G*Power version 3.1.9.7. The power analysis demonstrated that *n* = 204 was adequate (≥0.70) to identify a medium effect with an α level of *p* = 0.05. And we also describe the industry types of respondents to identify whether the composition of the industry type can represent the general situation in South Korea. The frequency analysis showed that the respondents were relatively evenly distributed across industries. In other words, this result indicates that the data in this study can be representative of the economic situation in South Korea (please see Table 1).

### 3.2. Measures

Research variables were measured using a five-point Likert scale (with scores ranging from 1 = strongly disagree to 5 = strongly agree).

#### 3.2.1. CSR (Gathered at Time Point 1, Gathered from Employees)

Drawing upon Turker’s CSR measurement [49] and additional CSR studies [50,51], the current research employed a 12-item CSR scale addressing various stakeholders, such as the environment, local communities, employees, and consumers. The Cronbach’s α value was 0.90.

#### 3.2.2. Ethical Climate (Time Point 1, Gathered from Employees)

The ethical climate was measured with four items of the Ethical Climate Questionnaire (ECQ) which was developed by Victor and Cullen [52]. ECQ measures members’ perceptions of ethical procedures and enforcement of codes in organizations.

The sample items were “If a person in my company is discovered to have engaged in unethical behavior that results primarily in corporate gain (rather than personal gain), she or he will be promptly reprimanded”; “The most important performance criteria for the employees of this organization are their compliance with the ethical rules and procedures”. The Cronbach’s α value was 0.84.

#### 3.2.3. POS (Time Point 2, Gathered from Employees)

The paper evaluated the employees’ degrees of POS using seven items from a previous study [40,41]. These answers were gathered during the second time point. These sample items included “there is someone who is truly considerate of me in my organization”, “there is a person who supports my growth and development in my organization”, and “my opinions are respected in my organization”. The Cronbach’s α value was 0.90.

#### 3.2.4. Organizational Commitment (Time Point 2, Gathered from Employees)

During Time Point 2, five items from the organizational commitment scale from an existing work were used [53]. The sample items included “I really feel as if my organization’s problems are my own”, “I feel a strong sense of belonging to my organization”, and “I feel emotionally attached to my organization”. The associated Cronbach’s α value was 0.92.

#### 3.2.5. Knowledge-Sharing Behavior (Time Point 3, Gathered from Employees’ Direct Supervisors)

This study used six items from the Knowledge-Sharing Behavior Scale [54]. Here, the level of knowledge-sharing behavior of each employee was assessed by their immediate supervisor. Sample items included “this employee explains everything very thoroughly” and “this employee tells me exactly what I need to know”. The Cronbach’s α value was 0.96.

#### 3.2.6. Control Variables (Time Point 2, Gathered from Members in an Organization)

To minimize estimation bias and enhance the validation of these findings, we controlled for the potential influence of gender, education, position, and employment tenure on knowledge-sharing behavior [54]. These variables were assessed at Time Point 1.

### 3.3. Analytical Approach

To appraise the validity of our research model, we employed SPSS version 26.0 and Amos version 26.0 (IBM Corp., Armonk, NY, USA) for executing various statistical analyses. Initially, the demographic attributes of the sample were examined using frequency analysis. Subsequently, the uniqueness of the measures was validated through confirmatory factor analysis. Pearson correlation analysis was applied to explore the relationships among study variables. To evaluate the hypotheses, structural equation modeling using Amos version 26.0 was deployed. In line with Anderson and Gerbing’s [55] recommendation, a two-step approach was implemented, encompassing (1) measurement and (2) structural modeling. To verify the measurement model, a confirmatory factor analysis was conducted. Thereafter, the AMOS version 26.0 program was employed to carry out a moderated mediation model analysis using the maximum likelihood estimator to examine the structural model via structural equation modeling. To establish the acceptability of diverse model fit indices, several goodness-of-fit indices, such as the comparative fit index (CFI), the Tucker–Lewis index (TLI), and the root mean square error of approximation (RMSEA), were employed. Previous research indicates that CFI and TLI values of >0.90 and an RMSEA value of <0.06 are acceptable benchmarks [56].

Lastly, a bootstrapping analysis was undertaken to gauge the significance of the indirect mediation effect between CSR activities and knowledge-sharing behavior with a 95% bias-corrected confidence interval (CI). The result of this analysis determines whether the indirect effect is statistically significant at a 0.05 level if the CI does not encompass “0” [57].

## 4. Results

### 4.1. Descriptive Statistics

This paper implemented correlation analysis to gain basic information about respondents. The research variables were all significantly related (please see Table 2).

### 4.2. Measurement Model

Confirmatory factor analyses were executed to evaluate the goodness-of-fit for the measurement model. The model included five research variables (CSR, POS and organizational commitment, ethical climate, and knowledge-sharing behavior), with its five-factor structure displaying a good fit (χ2 [df = 140] = 260.101, CFI = 0.978, TLI = 0.973, and RMSEA = 0.046). A sequence of chi-squared difference tests was subsequently conducted, comparing the four-factor model to alternative models with four factors (χ2 [df = 144] = 657.354, CFI = 0.905, TLI = 0.887, and RMSEA = 0.093), three factors (χ2 [df = 147] = 1206.142, CFI = 0.803, TLI = 0.771, and RMSEA = 0.133), two factors (χ2 [df = 149] = 1543.321, CFI = 0.741, TLI = 0.702, and RMSEA = 0.151), and one factor (χ2 [df = 150] = 2788.581, CFI = 0.509, TLI = 0.441, and RMSEA = 0.207), respectively. The outcomes of the chi-squared difference tests indicate that the five-factor model has the best model fit in comparison to other alternative models, suggesting that the five variables possess a suitable degree of discriminant validity.

### 4.3. Structural Model

In this study, a moderated mediation model was formulated to explore the connection between CSR activities and knowledge-sharing behavior. The model encompassed (1) a mediating structure wherein the links between CSR and knowledge-sharing behavior were sequentially mediated by POS and organizational commitment and (2) a moderating structure in which the relationship between CSR activities and POS was moderated by the degree of ethical climate. To evaluate the potential multi-collinearity bias between CSR and ethical climate, variance inflation factors and tolerances were computed [58]. The findings indicated that both corporate social responsibility and ethical climate were not significantly impacted by the issue of multi-collinearity, as variance inflation factor scores (CSR = 1.005, ethical climate = 1.005) were <10 and tolerance scores were >0.2 (CSR = 0.995, ethical climate = 0.995).

#### 4.3.1. The Results of Mediation Analysis

To identify the most appropriate model, structural equation model analyses were executed, and a chi-squared difference test compared our proposed model with alternative models (i.e., a full mediation model and a partial mediation model). The full mediation model was identical to the partial mediation model, except for the direct path between CSR activities and knowledge-sharing behavior. Both models exhibited acceptable fit indices. The full mediation model (χ2 = 425.753 [df = 170], CFI = 0.948, TLI = 0.936, and RMSEA = 0.061) and the partial mediation model (χ2 = 411.255 [df = 169], CFI = 0.951, TLI = 0.939, and RMSEA = 0.059) demonstrated acceptable fit indices. However, the chi-squared difference test between the models (Δχ2 [1] = 14.498, *p* < 0.01) indicated that the partial mediation model was superior, suggesting that corporate social responsibility will influence knowledge-sharing behavior both “directly” and “indirectly” (via the sequential mediating effect of POS and organizational commitment) rather than only exerting a direct effect on it.

**Figure 2:**
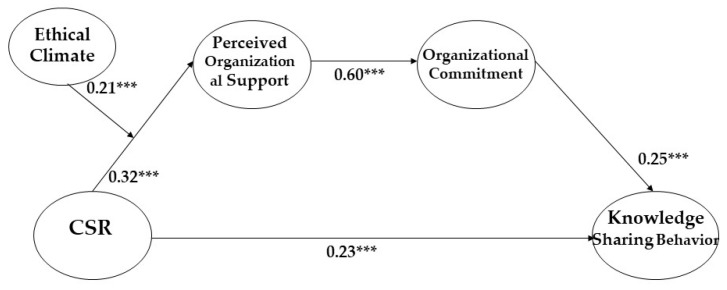
The final result of our research model with standardized values. *** *p* < 0.001.

All control variables (position, tenure, and education level) except for gender in this research model were not significant. All hypotheses were supported by the research model, including Hypothesis 1 that corporate social responsibility enhances knowledge-sharing behavior (β = 0.229, *p* < 0.001), Hypothesis 2 that CSR activities increase POS (β = 0.323, *p* < 0.001), Hypothesis 3 that POS enhances organizational commitment (β = 0.595, *p* < 0.001), and Hypothesis 4 that organizational commitment promotes knowledge-sharing behavior (β = 0.245, *p* < 0.001) (please refer to Table 3 and Figure 2).

Specifically, Hypothesis 1 was supported, as the path coefficient value between CSR activities and knowledge-sharing behavior in the partial mediation model was significant. This finding aligns with the enhanced model fit indices of the partial mediation model compared to the full mediation model. Therefore, we concluded that CSR activities would influence knowledge-sharing behavior both “directly” and “indirectly” through various mediators (e.g., POS and organizational commitment). Based on the results of the chi-squared difference test between the full and partial mediation models, as well as the significant path coefficient value, our findings support Hypothesis 1.

#### 4.3.2. The Result of Moderation Analysis

**Figure 3:**
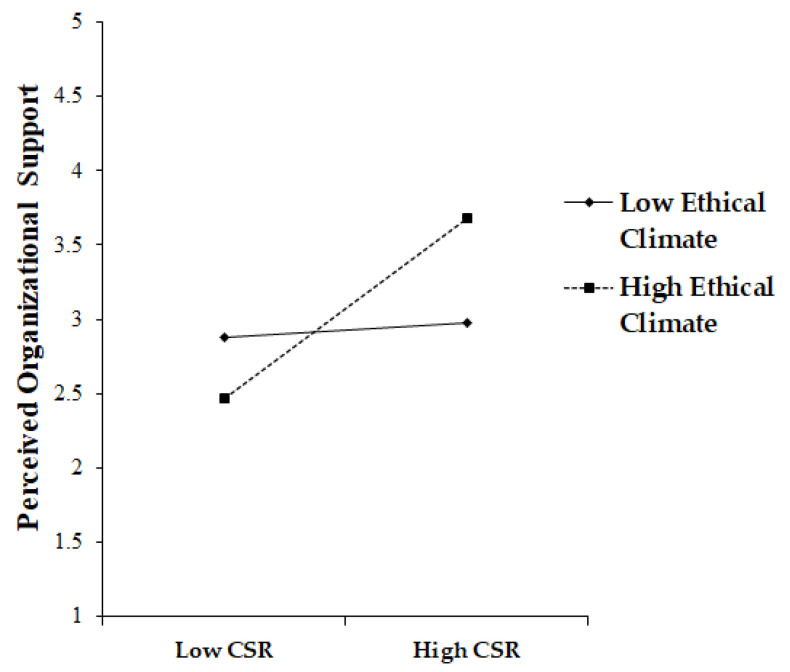
The moderating effect of ethical climate in the link between CSR and POS.

To examine the moderating influence of ethical climate on the relationship between CSR and POS, this investigation developed a moderated mediation model by generating an interaction term using a mean-centering technique. This approach facilitated the efficient estimation of interaction terms while diminishing multi-collinearity among the variables. The interaction term coefficient was determined to be statistically significant (β = 0.208, *p* < 0.001), indicating that ethical climate positively moderates the connection between CSR and POS. This result supported Hypothesis 6, as a higher level of ethical climate amplified the increasing effect of CSR on employee POS. Figure 3 offers a visual depiction of these findings.

### 4.4. Bootstrapping

To assess Hypothesis 5, which was a mediation hypothesis, a bootstrapping analysis employing a sample of 10,000 was conducted to ascertain the indirect mediation effect. The outcomes demonstrated that the bias-corrected CI for the average indirect effects on the paths excluded 0 (95% CI = [0.020, 0.092]), signifying support for Hypothesis 5. The direct, indirect, and overall effects of the paths from CSR activities to knowledge-sharing behavior can be found in Table 4.

## 5. Discussion

Drawing on various theoretical foundations, the present study investigates the relationship between CSR and employee knowledge-sharing behavior, examining the sequential mediation role of POS and organizational commitment as well as the moderating role of ethical climate. Six hypotheses were developed to explore the sequential mediation effect of POS and organizational commitment on the association between CSR and knowledge-sharing and the moderation effect of ethical climate on the connection between CSR and POS, respectively. To address potential common method bias issues, data were collected from 204 South Korean employees at three separate time points. The findings provide strong evidence that CSR activities positively impact employees’ knowledge-sharing behavior, an effect that is sequentially mediated by both POS and organizational commitment. Additionally, the study reveals the positive moderating role of ethical climate in the relationship between CSR and POS. By integrating multiple theoretical perspectives, this research not only contributes to the existing literature but also offers valuable insights for organizations aiming to enhance knowledge-sharing through the strategic implementation of CSR initiatives.

### 5.1. Theoretical Implications

The theoretical implications of this study on the nexus between CSR and employee knowledge-sharing behavior make a noteworthy contribution to the extant literature by addressing several research lacunae and proffering novel insights into the underlying mechanisms that sculpt this relationship. By scrutinizing the sequential mediation of POS and organizational commitment, this investigation advances our comprehension of the intricate interplay among CSR initiatives, employee perceptions, and knowledge-sharing behavior.

First, this research enriches the literature by incorporating the social identity theory, organizational citizenship behavior theory, and social cognitive theory to elucidate how CSR initiatives can bolster employees’ willingness to participate in knowledge-sharing behavior. By amalgamating these theoretical viewpoints, the study presents a comprehensive framework for understanding the impact of CSR on employees’ knowledge-sharing behavior (Hypothesis 1).

Second, this study extends the CSR literature by examining the mediating function of POS and organizational commitment in the CSR–knowledge-sharing nexus (Hypothesis 5). Drawing upon stakeholder theory and the conservation of resources theory, the investigation provides an innovative understanding of how CSR activities can augment employee POS (Hypothesis 2). Moreover, social exchange theory and the social identity theory are employed to explicate the connection between POS and employees’ organizational commitment (Hypothesis 3). These theoretical foundations enrich our grasp of the mediating mechanisms linking CSR initiatives with knowledge-sharing behavior.

Third, this research sheds light on the moderating capacity of ethical climate in the CSR–POS relationship (Hypothesis 6). By incorporating the social identity theory and social exchange theory, this study deepens our understanding of the boundary conditions that can either intensify or attenuate the influence of CSR initiatives on employee perceptions and attitudes.

### 5.2. Practical Implications

The practical implications of this study on the relationship between CSR and employee knowledge-sharing behavior furnish invaluable insights for top management teams and organizational leaders tasked with designing and implementing CSR initiatives that not only contribute to societal well-being but also cultivate a positive work environment that encourages employee knowledge-sharing.

First, this study underscores the importance of devising and executing CSR initiatives that effectively communicate the organization’s commitment to social responsibility. As the findings indicate, CSR activities can augment knowledge-sharing behavior among employees (Hypothesis 1) and POS (Hypothesis 2), which, in turn, can bolster employees’ organizational commitment (Hypothesis 3). By investing in substantive CSR initiatives, top management teams can engender a sense of pride and identification among employees, which can result in increased knowledge-sharing and collaboration within the organization.

Second, this research accentuates the role of POS in fostering employee commitment and knowledge-sharing behavior. Top management teams and leaders should be cognizant of the potential benefits of providing employees with adequate resources, support, and recognition for their contributions. By enhancing POS, organizations can fortify employees’ organizational commitment (Hypothesis 3) and stimulate knowledge-sharing behavior (Hypothesis 4).

Third, the study emphasizes the significance of cultivating an ethical climate within the organization. As the findings suggest, ethical climate positively moderates the relationship between CSR and POS (Hypothesis 6). Top management teams should prioritize the development of an ethical work environment that advocates transparency, fairness, and adherence to moral values. By fostering an ethical climate, organizations can further reinforce the positive effects of CSR initiatives on employee perceptions and attitudes.

In summary, the contributions of this study proffer practical insights for top management teams and leaders in organizations endeavoring to leverage the benefits of CSR initiatives. By concentrating on the design and implementation of efficacious CSR activities, enhancing POS, and nurturing an ethical work environment, organizations can promote employees’ commitment and knowledge-sharing behavior, ultimately contributing to a more innovative and collaborative organizational culture.

### 5.3. Limitations and Suggestions for Future Studies

Notwithstanding the considerable contributions of this investigation to the understanding of the interplay between CSR and employee knowledge-sharing behavior, it is imperative to recognize each study’s limitations and opportunities for future research.

First, we ought to consider the fact that the research sample was primarily sourced from South Korean companies, thus potentially preventing the applicability of the results to a wider array of cultural milieus. Consequently, in order to corroborate the findings and bolster their generalizability, subsequent inquiries should replicate the study across various settings, including an assortment of countries, industries, and organizational structures.

Second, the data collection method employed a self-reported survey, which might introduce common method bias. Future studies could consider using alternative data collection methods, such as observational data, supervisor evaluations, or secondary data sources, to verify the findings and mitigate potential biases.

Third, the study focused on the sequential mediation effect of POS and organizational commitment as well as the moderation effect of ethical climate. However, other potential mediators and moderators might also impact the relationship between CSR and knowledge-sharing behavior. Future research should explore additional variables, such as trust in the organization, psychological empowerment, or the role of leaders in shaping the relationship between CSR and knowledge-sharing.

Fourth, the study relied on cross-sectional data collected at three distinct time points, which may not fully capture the dynamic nature of the relationship between CSR and knowledge-sharing. Future studies could employ longitudinal or experimental designs to investigate the causal relationships between CSR initiatives, POS, organizational commitment, ethical climate, and knowledge-sharing behavior over time

Fifth, while the current study offers valuable insights into the interplay between CSR, ethical climate, employee perceptions, attitudes, and behaviors, its cultural context warrants particular attention. The research was conducted within a South Korean framework, a country with a distinct organizational culture characterized by high power distance, collectivism, and a strong emphasis on hierarchy [59]. These cultural facets may have influenced the observed interrelations between CSR, ethical climate, and knowledge behavior. For instance, collectivist cultures may exhibit different patterns of knowledge-sharing behavior compared to individualist cultures. Furthermore, the impact of CSR initiatives and the role of ethical climate could be perceived differently in cultures with varying degrees of power distance and uncertainty avoidance. As such, the findings’ direct applicability to other cultural contexts remains uncertain. Consequently, a limitation of the present research is the potential cultural-bound nature of the results, which should be acknowledged when considering its practical implications. To affirm the generalizability of the study’s findings, replication in diverse cultural settings is crucial. Comparative studies between different cultural contexts would allow for a broader understanding of how cultural dimensions interact with CSR, ethical climate, and employee perceptions/attitudes, thereby enriching the existing body of knowledge [60].

In addition, the study was conducted within a specific industry setting, which may have unique industry-specific dynamics that could influence the relationships observed. Future research would benefit from investigating these dynamics across various sectors. Comparisons between industries could uncover nuanced distinctions in how CSR and ethical climate interplay towards employee knowledge-related behavior [61]. Future research endeavors are encouraged to conduct similar investigations in diverse cultural and industry settings, which would provide a more comprehensive and globally applicable understanding of these complex relationships.

Sixth, although the present study brings to light the crucial roles of POS and organizational commitment as sequential mediators and ethical climate as a moderator, in the relationship between CSR and employee knowledge-sharing behavior, it is essential to acknowledge that other factors could also serve as mediators or moderators. The complexity of human behavior and organizational dynamics suggests that various psychological and organizational factors may significantly influence the observed relationships. One such potential mediator could be trust in the organization. Trust has been widely recognized as a critical element in knowledge-sharing behavior [62], and it is plausible that CSR initiatives could foster organizational trust, thereby enhancing knowledge sharing among employees. Future research could explore this potential mediating role of trust to provide a more nuanced understanding of the mechanisms through which CSR influences employee knowledge behavior. Psychological empowerment may serve as another potential mediator. Employees’ sense of empowerment, which can be fostered by CSR initiatives, might influence their inclination to share or withhold knowledge [63]. Studies could be conducted to explore how psychological empowerment might mediate the impact of CSR on knowledge behavior.

In terms of potential moderating variables, factors such as various types of leadership and individual characteristics (e.g., personality traits) could significantly influence the relationship between CSR and knowledge behavior [64]. For example, if the level of coaching leadership is high, the impact of CSR initiatives on employee knowledge behavior might be enhanced. Meanwhile, certain personality traits may make individuals more responsive to CSR initiatives which, in turn, might influence their knowledge sharing behavior. Future research should consider exploring these and other potential mediators and moderators to provide a more comprehensive understanding of the intricate dynamics among the variables. Such endeavors would contribute to both theoretical advancement and practical implications in the realm of CSR.

Lastly, despite the extensive insights provided by the present research, it is important to consider the inherent limitations of its design. Specifically, although we use 3-wave time-lagged research design, this paper cannot be free from the cross-sectional nature of the data collection from the fundamental point of view. Thus, the current paper may not fully encapsulate the dynamic and complex interplay among the research variables as well as the potential mediating and moderating factors. Given the likely time-lagged effects and the dynamic interplay among these variables, employing a cross-sectional design may present challenges in capturing the temporal sequencing and evolution of these relationships. This can potentially result in underestimation or overestimation of the effects of CSR on employee knowledge sharing behavior and the roles of POS, organizational identification, and ethical climate therein [65].

Therefore, future research could benefit significantly from employing longitudinal or experimental designs. Longitudinal designs allow for repeated measures of variables over time, thereby providing a more in-depth and precise understanding of the temporal sequences and potentially dynamic relationships among CSR, ethical climate, and employee knowledge-sharing behavior [66]. This approach would help to better elucidate the manner and degree to which changes in CSR initiatives influence changes in employee knowledge-related behavior and how these relationships are mediated and moderated by factors such as POS, organizational commitment, and ethical climate. Similarly, experimental designs, particularly field experiments, can provide a robust means to establish causal links among the variables [67].

## 6. Conclusions

In conclusion, this study contributes to our understanding of the relationship between CSR and employee knowledge-sharing behavior by scrutinizing the mediating roles of POS and organizational commitment as well as the moderating role of ethical climate. Drawing on several theoretical foundations, the study posits a comprehensive model that elucidates the intricate interplay between CSR initiatives and employees’ propensity to engage in knowledge-sharing behavior.

The findings of this research address several research gaps in the literature by clarifying the mechanisms through which CSR initiatives influence employee attitudes and behaviors. By demonstrating that POS and organizational commitment sequentially mediate the relationship between CSR and knowledge-sharing behavior (Hypothesis 5), this study advances our comprehension of the role of CSR in molding employees’ positive attitudes toward their organizations. Moreover, the study confirms the importance of ethical climate as a moderator in the CSR–POS relationship (Hypothesis 6), thus emphasizing the role of a conducive work environment in reinforcing the positive effects of CSR initiatives on employees’ perceptions.

## Figures and Tables

**Table 1:** Industry types of participants.

Industry Type	Percent
Manufacturing	20.4%
Services	14.7%
Construction	12.1%
Health and welfare	16.8%
Information services and telecommunications	8.0%
Education	16.3%
Financial/insurance	2.4%
Consulting and advertising	1.9%
Others	7.3%

**Table 2:** Correlation between variables.

	Mean	SD	1	2	3	4	5	6	7	8
1. Gender_T2	1.47	0.50	-							
2. Education_T2	2.70	0.84	−0.02	-						
3. Tenure_T2	67.92	73.99	−0.07	0.05	-					
4. Position_T2	2.42	1.51	−0.34 **	0.20 **	0.33 **	-				
5. CSR_T1	3.27	0.69	−0.05	0.22 **	0.16 **	0.07	-			
6. EC_T1	3.45	0.69	0.01	0.17 **	0.24 **	0.09	0.49 **	-		
7. POS_T2	3.15	0.80	−0.10	0.09	0.04	0.19 **	0.39 **	0.25 **	-	
8. OC_T2	2.95	0.92	−0.12 *	0.07	0.21 **	0.25 **	0.37 **	0.29 **	0.62 **	-
9. KSB_T3	2.82	0.90	0.07	0.12 *	0.09	0.08	0.32 **	0.29 **	0.34 **	0.28 **

Notes: * *p* < 0.05, ** *p* < 0.01. SD, standard deviation; CSR, corporate social responsibility; EC, ethical climate; KSB, knowledge-sharing behavior; OC, organizational commitment; POS, perceived organizational support.

**Table 3:** Correlation between variables.

Hypothesis	Path (Relationship)	Unstandardized Estimate	SE	Standardized Estimate	Supported
1	CSR → Knowledge-sharing Behavior	0.275	0.067	0.229 ***	Yes
2	CSR → POS	0.326	0.066	0.323 ***	Yes
3	POS → Organizational Commitment	0.680	0.058	0.595 ***	Yes
4	Organizational Commitment → Knowledge-sharing Behavior	0.256	0.054	0.245 ***	Yes
6	CSR × Ethical Climate	0.281	0.065	0.208 ***	Yes

Note: *** *p* < 0.001

**Table 4:** Direct, indirect, and total effects of the final research model.

Model (Hypothesis 4)	Direct Effect	Indirect Effect	Total Effect
CSR → POS → Organizational Commitment → Knowledge-sharing Behavior	0.229	0.047	0.277

All values are standardized. CSR, corporate social responsibility; POS, perceived organizational support.

## Data Availability

New data were created and analyzed in this study. Data sharing is not applicable to this article.
